# Cost-effectiveness analysis of the national implementation of integrated community case management and community-based health planning and services in Ghana for the treatment of malaria, diarrhoea and pneumonia

**DOI:** 10.1186/s12936-017-1906-9

**Published:** 2017-07-05

**Authors:** Blanca Escribano Ferrer, Kristian Schultz Hansen, Margaret Gyapong, Jane Bruce, Solomon A. Narh Bana, Clement T. Narh, Naa-Korkor Allotey, Roland Glover, Naa-Charity Azantilow, Constance Bart-Plange, Isabella Sagoe-Moses, Jayne Webster

**Affiliations:** 1 Disease Control Department, London School of Hygiene and Tropical Medicine, London, UK; 2 Dodowa Health Research Center, Ghana Health Service, Dodowa, Ghana; 3 Department of Public Health, University of Copenhagen, Copenhagen, Denmark; 4 School of Public Health, University of Health and Allied Sciences, Hohoe, Volta Region Ghana; 5 National Malaria Control Programme, Ghana Health Service, Accra, Ghana; 6 Reproductive and Child Health Department, Ghana Health Service, Accra, Ghana

**Keywords:** Home- based care, Integrated community case management (iCCM), Malaria, Diarrhoea, Pneumonia, Children under-five, Cost-effectiveness analysis

## Abstract

**Background:**

Ghana has developed two main community-based strategies that aim to increase access to quality treatment for malaria, diarrhoea and suspected pneumonia: the integrated community case management (iCCM) and the community-based health planning and services (CHPS). The aim of the study was to assess the cost-effectiveness of these strategies under programme conditions.

**Methods:**

A cost-effectiveness analysis was conducted. Appropriate diagnosis and treatment given was the effectiveness measure used. Appropriate diagnosis and treatment data was obtained from a household survey conducted 2 and 8 years after implementation of iCCM in the Volta and Northern Regions of Ghana, respectively. The study population was carers of children under-5 years who had fever, diarrhoea and/or cough in the last 2 weeks prior to the interview. Costs data was obtained mainly from the National Malaria Control Programme (NMCP), the Ministry of Health, CHPS compounds and from a household survey.

**Results:**

Appropriate diagnosis and treatment of malaria, diarrhoea and suspected pneumonia was more cost-effective under the iCCM than under CHPS in the Volta Region, even after adjusting for different discount rates, facility costs and iCCM and CHPS utilization, but not when iCCM appropriate treatment was reduced by 50%. Due to low numbers of carers visiting a CBA in the Northern Region it was not possible to conduct a cost-effectiveness analysis in this region. However, the cost analysis showed that iCCM in the Northern Region had higher cost per malaria, diarrhoea and suspected pneumonia case diagnosed and treated when compared to the Volta Region and to the CHPS strategy in the Northern Region.

**Conclusions:**

Integrated community case management was more cost-effective than CHPS for the treatment of malaria, diarrhoea and suspected pneumonia when utilized by carers of children under-5 years in the Volta Region. A revision of the iCCM strategy in the Northern Region is needed to improve its cost-effectiveness. Long-term financing strategies should be explored including potential inclusion in the National Health Insurance Scheme (NHIS) benefit package. An acceptability study of including iCCM in the NHIS should be conducted.

**Electronic supplementary material:**

The online version of this article (doi:10.1186/s12936-017-1906-9) contains supplementary material, which is available to authorized users.

## Background

In December 2014 a new global coalition of more than 500 leading health and development organizations worldwide was launched to urge governments to accelerate reforms that ensure everyone, everywhere, can access quality health services without being forced into poverty [1]. This global coalition, called the Universal Health Coverage (UHC), comprises two main components: quality essential health service coverage and financial coverage—both extended to the whole population [2].

With UHC on the global health agenda, governments of many low and middle-income countries are under pressure to scale up essential health services to meet the needs of their people. This means that governments need to prioritize effective interventions to scale up. Health technology assessments (HTAs) have been recognized as a tool for priority setting particularly useful in this UHC context [3]. HTA examines the cost-effectiveness of an intervention as well as the organizational implications and social consequences, bridging the gap between the evidence and policy making [4]. HTA can be used to prioritize an intervention in a particular context as mentioned before (ex-ante HTA) or it can be used as a monitoring tool, providing information to governments and funders to continue or discontinue certain interventions, helping to address sustainability (ex-post HTA) [3, 4].

Ghana has developed two main community based strategies that aim to reduce barriers to physical access to quality treatment: the iCCM and CHPS. Both strategies are implemented in the ten regions of Ghana through the Ghana Health Service and under the umbrella of the Ministry of Health. The iCCM strategy (then called home-based care) started on a pilot basis in Ghana in 1999 to treat suspected malaria cases [5]. The pilot programme initially used chloroquine, shifting to artemisinin-based combination therapy (ACT) in 2005 [6]. In 2009 and in the context of integrated management of childhood illness (IMCI), Ghana developed the Home Management of Malaria, ARI and Diarrhoea in Ghana [5]. iCCM was defined as prevention, early case detection and prompt and appropriate treatment of fevers, ARI and diarrhoea in the community. The iCCM strategy corresponds to the lowest level of health care delivery in Ghana and it is implemented through community-based agents (CBAs) selected by the community which received a 5-days training. The iCCM guidelines states that the service provided by the CBAs should be free [5] although some regions decided that users should give an small amount of money to CBAs to avoid risking continuity of the strategy. The HBC current funding is primarily reliant on external multilateral and bilateral donors as in many other countries [7–12]. The long-term financial plan for iCCM in Ghana is not clear.

The CHPS strategy started in 1999 after a pilot phase conducted in 1994 [13] attempting to respond to the 1978 Alma Ata Conference and the ‘Health for All’ principle. A key component of the CHPS strategy is that traditional leaders of the community must accept the CHPS concept and commit themselves to supporting it. The CHPS strategy is based upon a basic facility known as a community health compound, where health care is provided by a resident community health nurse or community health officer who also does a 90-day cycle visiting the communities she/he serves at least once within that period. Community health nurses receive formal training for a minimum of 2 years. Services provided by accredited CHPS are free for those having an active national insurance card.

After several years of national implementation, it is important to know how effective and cost-effective iCCM and CHPS are at delivering preventive and curative health services known to contribute to reducing the morbidity and mortality of children under-five. Studies that have looked at the effect of the iCCM in Ghana mostly focused in a few districts, looked particularly at the management of malaria home-based care, neglecting the cost-effectiveness and the preventive component, and were conducted in a more “controlled” context [14–18]. Ferrer et al. [19] reported on the utilization, effectiveness and users’ satisfaction of the curative component of iCCM and CHPS. This paper presents a cost-effectiveness analysis of iCCM versus CHPS. This study is an approach to ex-post HTA which considers the real costs and effectiveness of both interventions (iCCM and CHPS) which is key to policy decisions. Results from this study may be used to improve performance and to guide decisions on sustainable financing strategies particularly regarding iCCM.

## Methods

### Study site

The Volta and Northern Regions were purposively selected. Criteria for selecting these regions are described in Ferrer et al. [19]. In brief, the first criteria was the inclusion of a region implementing iCCM but providing only treatment for malaria and a region providing treatment for malaria, diarrhoea and pneumonia, to have a better picture of the iCCM strategy in Ghana. The second criteria was different implementation performance levels, based on the routine information system. The CHPS strategy is uniform across all ten regions of the country. The iCCM in the Volta Region was supported mainly through the Global Fund and targeted only rural districts for the iCCM implementation. Volta Region had a population of 1,901,179 habitants, 17 districts, 674 CHPS and 920 CBAs in 2014; malaria prevalence was 17%, diarrhoea prevalence was 7.6% and suspected pneumonia prevalence was 2.1% in children under-five [20]. The rural population corresponded to 66% of the total population. Two rainfall seasons occur in the middle and coastal belts. One major season is in April/July with a peak in June and one minor season is in September/November with a peak in October. The north of Volta Region has one rainy season—May to October with a peak in August.

The iCCM in the Northern Region was supported mainly through UNICEF and targeted all communities. It had a population of 2,479,461 habitants, 20 districts, 210 CHPS and 5000 CBAs in 2014; malaria prevalence was 48%, diarrhoea prevalence was 21.4% and suspected pneumonia prevalence was 6.3% in children under five [21]. The rural population corresponded to 70% of the total population. In the north, the rainy season begins in May and ends in October [22]. Climatically, religiously, linguistically, and culturally, the Northern Region differs greatly from the politically and economically dominating regions of central and southern Ghana, and it is similar to the two other northern regions (Upper East and Upper West).

### Measurement of effect

The effectiveness measure used was ‘case appropriately diagnosed and treated’. This refers to a malaria, diarrhoea or suspected pneumonia case that received treatment according to treatment guidelines (Table 1) or a child without malaria, diarrhoea or suspected pneumonia that was not prescribed the recommended drugs to treat malaria, diarrhoea or pneumonia. Definitions of a malaria, diarrhoea and suspected pneumonia case are presented in Table 1. Appropriate diagnosis and treatment is a proxy indicator of child mortality. Malaria cases can progress rapidly to complication and death if malaria treatment is not administered in the first 24–48 h from onset of symptoms [23]. Prompt treatment with a full course of effective antibiotics is key to reduce pneumonia deaths [24]. ORS and zinc are effective therapeutic interventions to reduce diarrhea mortality [25].

**Table 1:** Study definitions and treatment guidelines

Definitions	iCCM [5]	CHPS [52]
Malaria	All fever cases when no laboratory tests are available	All fever cases when no laboratory tests are available or when malaria test was positive
Appropriate treatment of malaria	Children aged 6 months to 5 years diagnosed with malaria receiving 3 days of ACT	Children aged 2 months to 5 years diagnosed with malaria receiving 3 days of ACT
	If more than 7 days with fever, general danger signs or severe malaria signs, child must be referred with rectal artesunate	If more than 7 days with fever, general danger signs or severe malaria signs, child must be referred with IM quinine, IM or EV or rectal artesunate plus an IM dose of chloramphenicol
Prompt treatment of malaria	Malaria cases that received an antimalarial drug in within the first 24 h of the onset of symptoms	The same definition as in iCCM
Diarrhoea	3 or more loose or watery stools in a 24-h period	The same definition as in iCCM
Appropriate treatment of diarrhoea	Children older than 6 months with diarrhoea of less than 7 days that receive ORS and zinc for 14 days	Children with diarrhoea of less than 14 days receiving ORS and zinc for 14 days
	If the child is less than 6 months, had diarrhoea for 7 days or more, blood in stools or is dehydrated, he/she should be referred with ORS	If diarrhoea for 14 days or more, blood in stools or is severely dehydrated, he/she should be referred to hospital with ORS
ARI or suspected pneumonia	Cough with fast or difficult breathing^a^	The same definition as in HBC^b^
Appropriate treatment for suspected pneumonia	Children older than 6 months with cough and fast or difficult breathing of less than 7 days receiving amoxicillin for 5 days	Children older than 2 months with cough and fast or difficult breathing of less than 14 days receiving amoxicillin or co-trimoxazol for 5 days
	If the child is less than 6 months or had symptoms for 7 days or more, he/she should be referred	If the child is less than 2 months or had symptoms for 14 days or more, he/she should be referred
	If there are signs of severe pneumonia, he/she should be referred with amoxicillin	If there are signs of severe pneumonia, he/she should be referred with IM chloramphenicol

^a^ARI timers are available in the Northern Region under the iCCM strategy to help diagnose suspected pneumonia. If severe pneumonia is suspected, the child must be referred to a CHPS compound or a Health Centre

^b^Nurses at CHPS compounds do not have ARI timers. The diagnosis is made based on clinical signs. If a severe pneumonia case is suspected, the children must be referred to a higher level of health facility. Some district hospitals, all regional hospitals and teaching hospitals have X-rays to help diagnose pneumonia. Health centres, district hospitals, regional hospitals and teaching hospitals have laboratory facilities to help diagnose malaria, diarrhoea and pneumonia

Data from a cross sectional household survey was used to assess the number of cases appropriately diagnosed and treated under the iCCM and CHPS [19]. In brief, a stratified three stage cluster survey was conducted in the Volta and Northern Regions. In order for the sample to be representative of the whole region, whilst being logistically feasible, regions were divided into three areas. From each area, two districts and from each district four clusters were selected using probability proportional to size. Then, from each cluster 27 households were selected, making a total of 648 households in each region. To select the districts (first stage) the list of districts implementing HBC (all districts implement the CHPS strategy) with its population was used. According to the NMCP, from the 24 districts in the Volta Region, only eight were targeted for the implementation of iCCM and these comprised the sampling frame. In the Northern Region all districts and communities were included in the sampling frame, as iCCM was implemented everywhere. To select the clusters (second stage) the list of communities implementing iCCM with its population was used. Then, households with children under-five that had fever, diarrhoea or cough in the last 2 weeks prior to the interview were randomly selected in each cluster using a modified expanded programme on immunizations sampling technique (third stage) [26]. To select households, a location near the centre of the community was first identified and a random direction was defined by spinning a pen. A random household along the chosen direction pointing outwards from the centre of the community to its boundary was chosen and checked for compliance with the inclusion and exclusion criteria. Whether the criteria were met or not, the next closest household was visited until the required number of households with a child with a fever, diarrhoea or cough in the 2 weeks preceding the survey were surveyed. Interviews were conducted with the carer of the sick child. In cases where there was more than one eligible child in a household, only one of them was selected randomly by ballot paper.

Data collection was done using a structured questionnaire which included socio-demographic information of the carer, care-seeking behaviour, treatment received and source of treatment, experience with health providers and costs involved when seeking care.

### Measurement of costs

Economic costing was done from the societal perspective, which considers costs from the perspective of households and the health system. The societal perspective is broader than the health system or government budget perspective [27], and allows comparison with previous studies. It is important to consider household costs because they can be significant, they may deter caregivers from using a service and they might be a cause of poverty (catastrophic cost). Data on cost was collected from the household survey, the Health Administration and Support Services division (HASS) of the Ghana Health Service, the National Malaria Control Programme, CHPS and from the NGO Plan International which supports HBC in the Volta Region. Unit cost was defined as the total cost incurred in diagnosing and treating one case of malaria, diarrhoea or suspected pneumonia.

Two activities were used to calculate the cost per case diagnosed and treated: (1) estimation of unit cost for treating a malaria, diarrhoea and suspected pneumonia case from the iCCM, CHPS, and households and (2) combination of unit cost for treating a malaria, diarrhoea and suspected pneumonia case under the iCCM and CHPS strategies with the household costs to obtain unit cost from the societal perspective.

### Estimation of unit costs for treating a malaria, diarrhoea and suspected pneumonia case from the iCCM, CHPS and households

#### iCCM costs

Costs per diagnosing and treating a malaria, diarrhoea and suspected pneumonia case under the iCCM strategy were divided into direct and indirect costs. Direct costs referred to those involved directly in the service. Indirect costs referred to productivity loses due to the time a CBA spent away from their usual activities (attending a sick child). Direct recurrent costs included training of CBAs (according to policy it happens every 2 years), training of supervisors (according to policy it happens every 2 years), supervision (every year), training material for supervisors and CBAs and IEC material for CBAs (last several years), registers (every year) and cost of drugs (every year). Direct capital costs included ARI timers (to measure the breath rate to diagnose suspected pneumonia cases), box to keep CBA items and the incentive package (includes bicycles, raincoats, boots, torchlights and t-shirts) which, according to policy, is given once at the beginning of the intervention to motivate CBAs.

With regards to the Volta Region, Plan International provided information on cost for training CBAs and supervisors. Training expenditures were annualized using the expected life time of 2 years and the recommended discount rate of 3%. Annualizing capital costs means that even though a good has been purchased at a specific point in time, one needs to consider that its benefits will be enjoyed over several years, and therefore, its costs will be spread over these years. The directorate of the Volta Region provided information on the quantities of training material, registers, boxes and incentive package received from the national level, while the national level provided information about the cost per item sent to the regions. A 10% of freight costs were added to the unit costs. The training and IEC materials, the boxes and the incentive package are supposed to last several years, and therefore, they were annualized using the expected life time of 8 years and the discount rate of 3% [31]. The NMCP reported no ARI timers in the Volta Region. The number of drugs used in 2014 was obtained from the DHIMS2. The cost of drugs used was the median price from a list of suppliers obtained from the International Drug Price Indicator Guide [36] with an addition of 10% freight costs as when calculating CHPS costs.

The regional directorate of the Northern Region provided information about costs of training CBAs and supervisors. Training expenditures were annualized using the expected life time of 2 years and the recommended discount rate of 3% [31]considering that, according to policy, these trainings happen every 2 years. The directorate of the Northern Region provided information on the quantities of training and IEC material, registers, boxes and incentive package received from the national level, while the national level provided information about the cost per item sent to the regions. To these item cost, a 10% of freight costs were added. The training materials, the boxes and the incentive package were annualized using the expected life time of 8 years and the recommended discount rate of 3% as I did for the Volta Region. The unit cost of ARI timers used was US$3.5 plus a 10% of freight costs as recommended by UNICEF. The number of drugs used in 2014 was obtained from the DHIMS2. The cost of drugs used was the median price from a list of suppliers obtained from the International Drug Price Indicator Guide [36] with an addition of 10% freight costs as when calculating CHPS costs and iCCM costs in the Volta Region.

As indirect cost, the time of the CBAs attending a sick child was included. The opportunity cost to their time was estimated from interviews with four CBAs, two in each region. CBA time was assigned a monetary value based on the agricultural labour wage the main occupation of the population, particularly in rural areas [28]—which was 0.41 GHC/h [29, 30]. One GHC was equivalent to US$ 0.31 in 2014. All costs were estimated for 2014 and were converted to US dollars. Direct and indirect costs were allocated to malaria, diarrhoea and suspected pneumonia case management based on the percentage of iCCM activity reported in 2014 through the DHIMS-II.

#### CHPS costs

Two CHPS compounds were visited in each region to collect information during the 5th to 16th April 2014 in the Volta Region and during the 23rd June to 3rd July 2014 in the Northern Region. The criteria used to select these facilities was average performing facilities based on their activity (outpatient visit per nurse). This information was provided by the regional directorates. In addition, as cost information was collected during the household survey, the criteria of feasibility was also considered. This means that to select two CHPS among average CHPS, those that were closer to the communities being surveyed were considered. Two CHPS compounds were selected in Hohoe district (Volta Region), one in Central Gonja district (Northern Region) and one in Tolon-Kumbugu district (Northern Region).

Direct cost were considered from the CHPS perspective, including recurrent and capital costs (Table 2). As recurrent cost, salaries of personnel including any allowances received, medicines, disposables, stationary, utilities and maintenance costs were considered. 2014 recurrent expenditures were obtained from the financial officer at the respective health districts. Capital cost were the building, vehicles, furniture and equipment that were annualized using the expected life time of 30, 8, 10 and 8 years, respectively, and the recommended discount rate of 3% [31]. The size of the CHPS facilities was estimated based on plans of standard CHPS facilities available from the HASS division. The information on construction cost per m^2^ for such buildings was obtained from the same division. An inventory of equipment and furniture was developed during the field visits at the two CHPS in each region. These items were valued using a price list from the HASS division. If the cost of an existing equipment or furniture was not in the list provided by the HASS division, market surveys were conducted to value these items. To do this, the average of a sample of at least three different prices were considered for one equipment or furniture as suggested in the guidelines for cost data collection in the field in ACT consortium projects [32]. These sample prices were mainly obtained online in the case of equipment and in Ghanaian shops in the case of furniture. All costs were estimated for 2014 (taking into account the inflation rates when needed) and were converted to US dollars.

**Table 2:** Cost per diagnosing and treating a malaria, diarrhoea and suspected pneumonia case under the iCCM strategy in the Volta and Northern Region in 2014 (US$)

	Volta Region			Northern Region		
	Malaria	Diarrhoea	Pneumonia	Malaria	Diarrhoea	Pneumonia
Programme costs						
Training CBAs	881.43	125.57	40.73	18,756.66	25,024.98	9579.92
Training supervisors	446.84	63.66	20.65	7062.53	9422.77	3607.17
Supervision	0.00	0.00	0.00	1473.76	1966.28	752.72
Training and IEC materials	60.93	8.68	2.82	1069.25	1426.58	546.11
CBA drugs	36,088.88	837.48	1266.76	12,698.00	4343.24	5966.96
ARI timers	0.00	0.00	0.00	0.00	0.00	658.15
Registers	430.25	61.29	19.88	5961.00	7953.11	3044.57
Time of CBAs	1426.16	210.89	68.40	484.11	645.90	247.26
Drugs box	86.39	12.31	3.99	1448.60	1932.72	739.87
Incentive package	1167.01	166.25	53.92	20,478.34	27,322.03	10,459.27
Total programme costs	40,587.89	1486.12	1477.14	69,432.25	80,037.61	35,602.01
Average cost per case	1.54	0.38	1.12	7.77	6.72	7.80

The allocation of recurrent and capital costs to outpatient visits was performed using the standard step down costing methodology [33]. First, all resources were allocated to all cost centres using different allocation criteria suggested in the guidelines of ACT consortium projects [32] (step 1). Then, the overhead costs were allocated to support and final centres also using relevant allocation criteria (step 2). Finally, support centres costs were allocated to outpatient visits (step 3).

To differentiate cost per malaria, diarrhoea and suspected pneumonia treatment in under-fives from the rest of the outpatient visits, a bottom up costing method was conducted. Interviews with staff captured self-reported time for doing a RDT and treating a case of malaria, diarrhoea and suspected pneumonia in children under-5 years of age. The average cost of RDT was taken from two market surveys by the Global Fund and the Programme for Appropriate Technology in Health (PATH) [34, 35] which gave an average cost of US$0.35 plus a 10% of freight costs. Details of the drugs given were taken from a sample of patients (between 6–12 cases per disease in each CHPS). The cost of drugs used was the median price from a list of suppliers obtained from the International Drug Price Indicator Guide [36] with an addition of 10% freight costs as recommended by the guide.

#### Household costs

Household costs incurred when visiting a CBA or a CHPS facility were also divided into direct and indirect cost. Direct costs included cost per transport to provider and any other direct cost incurred at the provider such as drug costs or diagnostic costs. Indirect costs related to productivity loses and referred to time travelling to a CBA/CHPS and time spent at/with the provider. These costs were obtained from the household survey conducted in 2014. Cost of carers time was calculated based on the agricultural labour wage [29, 30].

These household costs were obtained from the household survey conducted in 2014. Carers seeking care when their child was sick, were asked about all the different cost involved based on the different provider they visited. Therefore, household costs when visiting a CBA or a CHPS (or any other provider) were different and were accounted in the analysis.

### Estimation of unit costs for treating a malaria, diarrhoea and suspected pneumonia case under the HBC and CHPS strategy from the societal perspective

Facility and programme costs were added to the household costs to obtain costs from the societal perspective under the HBC and CHPS strategies. Household costs related to drugs or RDT were also considered (even if they were already included when analysing the unit cost for the management of malaria, diarrhoea and suspected pneumonia from the HBC and CHPS perspective). Although the difference between including or excluding these costs was very little, as they are an extra cost for the household, it was decided to include them in the final cost.

Data from the survey showed that no deaths were reported after visiting a CBA or a CHPS in the Volta and Northern Regions. Reported referrals after visiting a CBA were 14.1 and 20.1% in the Volta and Northern Region, respectively, and 5.9 and 4.3% after visiting a CHPS compound. However, 42 and 63% of carers sought care elsewhere after using iCCM and 28 and 7.9% after using CHPS in the Volta and Northern Regions, respectively. This high proportion of carers visiting a second provider (particularly for the iCCM strategy) is important as it represents an extra cost to diagnose and treat a case. Therefore, costs due to this second visit to a provider were also taken into consideration and added to the unit cost for the diagnosis and management of malaria, diarrhoea and suspected pneumonia. To do this, the proportion of carers that sought care to a health facility or to a licensed chemical seller because of malaria, diarrhoea or suspected pneumonia was multiplied by the cost of a second visit. The cost of the second visit was calculated by adding programme/facility costs to household costs. This included: (1) the average of iCCM cost for diagnosing and treating a malaria, diarrhea and suspected pneumonia (if second provider was a licensed chemical seller or a drug peddler), or CHPS cost (if second provider was a health facility); (2) the average cost for traveling to the second provider and (3) the average money spent at the second provider visited. Taking into account that CHPS were the health facility more often used in this second visit, and that expenditures from other health facilities, from licensed chemical sellers or from drug peddlers were not available, it was believed that using CHPS and CBA cost was the best approach to calculate the cost for diagnosis and treating a case in the second visit.

### Measurement of the cost-effectiveness

The appropriate comparison of costs and effects between two programmes or interventions is the incremental cost-effectiveness ratio (ICER) [33]. In this study, rather than presenting ICERs numerically, the results were presented with the aim of helping to guide policy makers. Based on the cost-effectiveness plane [33], the following classifications were used to explore in which circumstances it might be appropriate to support the HBC strategy with public funds:

- iCCM dominates: iCCM is less costly and more effective.
- iCCM is more costly and more effective.
- iCCM is less costly and less effective.
- iCCM is dominated: iCCM is more costly and less effective.

From a policy-maker’s perspective, if iCCM dominates it would justify the support of this strategy with public funds, without necessarily saying that the CHPS strategy should be discontinued (as iCCM aims to complement CHPS—and not to substitute them—as a strategy to increase access to quality treatment). If iCCM is more costly and more effective, the decision on financing iCCM or not will depend on the government’s willingness to pay, as there is no threshold to suggest how much extra money is reasonable to pay per extra case appropriately treated (while there is a suggested threshold on cost per DALYs averted or QALY gained that considers an intervention “highly attractive” or cost-effective [37]). If iCCM is less costly but less effective, it is not likely to be considered worthwhile using public funds unless the difference in effectiveness is minimal. If iCCM is dominated, this suggests that the intervention should not be supported as it is currently being implemented.

### Sensitivity analysis

To deal with uncertainty in this study a one-way sensitivity analysis was conducted. The parameters chosen for the sensitivity analysis were discounting rates (3, 5 and 7%), facility costs (average, lower and higher costs), different degrees of iCCM and CHPS utilization (based on the limits of the 95% CI obtained from the household survey) and iCCM effectiveness (50% increase and decrease).

## Results

Calculation of the unit costs for diagnosing and treating a malaria, diarrhoea and suspected pneumonia case in children under-5 years of age from the health system, household and societal perspective is presented, followed by the cost-effectiveness and sensitivity analyses.

### Unit cost for treating a malaria, diarrhoea and suspected pneumonia case from iCCM, CHPS, and households

Average iCCM programme costs in the Volta Region were lower when compared with those of the Northern Region: diagnosing and treating a malaria case costs US$1.54 and US$7.77, a diarrhoea case costs US$0.38 and US$6.72 and a suspected pneumonia case cost US$1.12 and US$7.80 in the Volta and Northern Region, respectively (Table 2). The much higher iCCM costs in the Northern Region when compared with those of the Volta Region was mainly due to (1) higher costs for training and for the incentive package in the Northern Region (due to a higher number of CBAs); (2) a lower number of visits to sick children in the Northern Region (17,898 and 30,839 visits in the Northern Region and Volta Region) and (3) a much lower number of IEC activities (177,484 and 99 IEC activities conducted in the Volta Region and the Northern Region).

Across the Volta and the Northern Regions, the cost per diagnosing and treating a malaria case in a CHPS varied from US$4.65 to US$9.95; the cost per treating a diarrhoea case varied from US$3.05 to US$8.16 and the cost per treating a suspected pneumonia cased varied from US$3.11 to US$8.79 (Table 3). When compared with the Northern Region, the Volta Region had the lower and higher costs per malaria, diarrhoea and suspected pneumonia case diagnosed and treated across both regions. These differences in the Volta Region were mainly due to the existence of a bigger facility with smaller reported activity compared to a smaller facility with higher reported activity. In the Northern Region, the micro-costing exercise showed that the large differences observed in diagnosing and treating a malaria case in the two CHPS sampled were mainly due to the use of ACT syrup in one of the CHPS (which cost US$ 0.1/ml) instead of ACT tablets (which costs US$ 0.01/tablet).

**Table 3:** Cost per diagnosing and treating a malaria, diarrhoea and suspected pneumonia case in four CHPS facilities in the Volta and Northern Region in 2014 (US$)

	Volta Region						Northern Region					
	CHPS 1			CHPS 2			CHPS 1			CHPS 2		
	Malaria	Diarrhoea	Pneumonia	Malaria	Diarrhoea	Pneumonia	Malaria	Diarrhoea	Pneumonia	Malaria	Diarrhoea	Pneumonia
Recurrent expenditures												
Salaries	625.15	338.48	43.72	582.09	119.40	104.48	745.52	342.01	9.84	1386.98	290.05	7.91
Stationery	9.15	4.96	0.64	4.84	0.99	0.87	2.16	0.99	0.03	56.08	11.73	0.32
Utilities	0.00	0.00	0.00	2.04	0.42	0.37	0.00	0.00	0.00	2.24	0.47	0.01
Training	12.46	6.75	0.87	3.02	0.62	0.54	54.83	25.15	0.72	35.33	7.39	0.20
Capital expenditures												
Buildings	15.73	8.52	1.10	34.81	7.14	6.25	12.86	5.90	0.17	17.55	3.67	0.10
Equipment	54.30	29.40	3.80	9.41	1.93	1.69	82.78	37.97	1.09	18.06	3.78	0.10
Furniture	7.32	3.96	0.51	10.43	2.14	1.87	4.15	1.90	0.05	1.82	0.38	0.01
Overhead												
Administration	37.32	20.21	2.61	86.54	17.75	15.53	30.75	14.11	0.41	60.82	12.72	0.35
Accounting	10.41	5.64	0.73	92.10	18.89	16.53	15.41	7.07	0.20	118.90	24.86	0.68
Health information	20.38	11.04	1.43	113.58	23.30	20.39	10.36	4.75	0.14	345.80	72.32	1.97
Cleaning	71.27	38.59	4.99	61.16	12.55	10.98	7.48	3.43	0.10	26.90	5.63	0.15
Laundry	0.00	0.00	0.00	0.00	0.00	0.00	0.00	0.00	0.00	47.40	9.91	0.27
Stores	28.22	15.28	1.97	26.38	5.41	4.73	0.00	0.00	0.00	134.51	28.13	0.77
Security	0.00	0.00	0.00	52.08	10.68	9.35	0.00	0.00	0.00	0.00	0.00	0.00
Support centres												
Dispensary drugs	499.06	115.27	16.52	33.07	19.91	35.23	365.31	102.18	7.42	1739.20	99.70	4.30
Dispensary overhead	71.98	38.97	5.03	96.70	19.84	17.36	0.00	0.00	0.00	111.46	23.31	0.64
Diagnostics	330.36	0.00	0.00	344.65	0.00	0.00	109.81	0.00	0.00	273.12	0.00	0.00
Total	1793.12	637.06	83.93	1552.90	260.97	246.16	1441.42	545.47	20.17	4376.15	594.04	17.79
Average cost per case	4.65	3.05	3.11	9.95	8.16	8.79	4.76	3.92	5.04	8.32	5.40	5.93

Diagnosing and treating malaria was found to be more costly than diarrhoea or suspected pneumonia in 3 out of 4 CHPS facilities. All three diseases needed similar resources in terms of building, furniture and equipment. However, in the case of malaria the cost increased because of the time invested in doing the RDT and the laboratory costs. In addition, and as mentioned above, the cost of ACT syrup was higher than the cost of ACT tablets and higher than ORS (0.07/sachet), zinc (0.02/tablet) or amoxicillin (0.004/ml). Household costs represent the cost of time used to travel to the provider, the money spent on travel, the cost of time spent with the provider, the money spent at the provider in terms of food and other costs involved such as paying for the service or for drugs. As expected, household costs were higher when visiting a CHPS facility than when visiting a CBA, particularly because of longer distances from households to a CHPS facility than to a CBA, higher cost of transport and longer time at a CHPS facility than with a CBA. Household costs ranged from US$0.04 when visiting a CBA in the Northern Region (corresponding to a 0.4% of the total cost per case) to US$1.54 when visiting a CHPS in the Volta Region (corresponding to 20.6% of the total cost per case) (Table 4).

**Table 4:** Cost per diagnosing and treating a malaria, diarrhoea and suspected pneumonia cases from the household perspective under the iCCM and CHPS strategy in the Volta and Northern Region in 2014 (US$)

Variables	Volta Region						Northern Region					
	CBA			CHPS			CBA			CHPS		
	Malaria	Diarrhoea	Pneumonia	Malaria	Diarrhoea	Pneumonia	Malaria	Diarrhoea	Pneumonia	Malaria	Diarrhoea	Pneumonia
Value of time lost to provider (1)	0.03	0.03	0.03	0.12	0.12	0.12	0.02	0.02	0.02	0.08	0.08	0.08
Travel cost (2)	0.00	0.00	0.00	1.10	1.10	1.10	0.00	0.00	0.00	0.18	0.18	0.18
Value of time lost at provider (3)	0.03	0.03	0.03	0.05	0.05	0.05	0.02	0.02	0.02	0.07	0.07	0.07
Food in provider (4)	0.01	0.01	0.01	0.22	0.22	0.22	0.00	0.00	0.00	0.35	0.35	0.35
RDT cost (5)	0.00	0.00	0.00	0.07	0.00	0.00	0.00	0.00	0.00	0.10	0.00	0.00
Drug cost (6)	0.01	0.03	0.00	0.25	0.05	0.00	0.00	0.08	0.00	0.12	0.03	0.54
Average cost per case	0.07	0.09	0.06	1.81	1.54	1.49	0.04	0.11	0.04	0.91	0.72	1.24

(1) Refers to the value of time carers spent travelling to provider to sick care for their children; (2) Money spent on transport to provider by carers when seeking care; (3) Value of time spent at provider by carers when seeking care; (4) Money spent by carers buying food at provider when seeking care for their children; (5) Money spent paying for RDT; (6) Money spent paying for drugs

### Unit cost for treating a malaria, diarrhoea and suspected pneumonia case from the societal perspective

To obtain costs from the societal perspective, household costs were added to programme costs of the iCCM and CHPS strategies (Table 5).

**Table 5:** Cost per diagnosing and treating a malaria, diarrhoea and suspected pneumonia case from the societal perspective under the iCCM and CHPS strategy in the Volta and Northern Region in 2014 (US$)

	Volta Region						Northern Region					
	CBA			CHPS			CBA			CHPS		
	Malaria	Diarrhoea	Pneumonia	Malaria	Diarrhoea	Pneumonia	Malaria	Diarrhoea	Pneumonia	Malaria	Diarrhoea	Pneumonia
Provider perspective												
Programme cost per case	1.54	0.38	1.12	7.30	5.60	5.95	7.77	6.72	7.80	6.54	4.66	5.49
If second provider was sought	3.34	0.41	0.14	0.41	0.11	0.02	1.56	1.53	0.66	0.61	0.16	0.00
Total facility/programme cost per case	4.88	0.79	1.26	7.71	5.71	5.97	9.33	8.24	8.46	7.15	4.82	5.49
Household perspective												
Value of travel time lost to provider	0.03	0.03	0.03	0.12	0.12	0.12	0.02	0.02	0.02	0.08	0.08	0.08
Travel cost	0.00	0.00	0.00	1.10	1.10	1.10	0.00	0.00	0.00	0.18	0.18	0.18
Value of time lost at provider	0.03	0.03	0.03	0.05	0.05	0.05	0.02	0.02	0.02	0.07	0.07	0.07
Food in provider	0.01	0.01	0.01	0.22	0.22	0.22	0.00	0.00	0.00	0.35	0.35	0.35
RDT	0.00	0.00	0.00	0.07	0.00	0.00	0.00	0.00	0.00	0.10	0.00	0.00
Drugs	0.01	0.03	0.00	0.25	0.05	0.00	0.00	0.08	0.00	0.12	0.03	0.54
Total household cost per case	0.07	0.09	0.06	1.81	1.54	1.49	0.04	0.11	0.04	0.91	0.72	1.24
Average cost per case	4.96	0.88	1.33	9.52	7.25	7.45	9.37	8.36	8.50	8.07	5.54	6.73

Costs involved due to a second visit to a provider after visiting a CBA or a CHPS facility were added to programme/facility costs. In the Volta Region, average costs for this second visit were US$3.34 and US$0.41 per malaria case treated under the iCCM and CHPS strategy respectively; US$0.41 and US$0.11 per diarrhoea case treated under the iCCM and CHPS strategy and US$0.14 and US$0.02 per suspected pneumonia case treated under the iCCM and CHPS strategy respectively. In the Northern Region, average costs for the second visit to a provider were US$1.56 and US$0.61 per malaria case under the iCCM and CHPS strategy respectively; US$1.53 and US$0.16 per diarrhoea case treated under the iCCM and CHPS strategy and US$0.66 and US$0.001 per suspected pneumonia case treated under the iCCM and CHPS strategy, respectively.

After adding household costs and those of the second visit to a provider, the cost per diagnosing and treating a malaria case under the iCCM strategy was US$4.96 and US$9.37 in the Volta and Northern Regions and US$9.52 and US$8.07 in a CHPS in the Volta and the Northern Regions; to treat a diarrhoea case costs US$0.88 and US$8.36 under the iCCM in the Volta and Northern Regions and US$7.25 and US$5.54 in a CHPS in the Volta and the Northern Regions. Finally, treating a suspected pneumonia case cost US$1.33 and US$8.50 under the iCCM strategy in the Volta and the Northern Regions and US$7.45 and US$6.73 in a CHPS in the Volta and the Northern Regions, respectively.

### Cost-effectiveness analysis

Tables 6, 7 and 8 presents the average cost per case appropriately diagnosed and treated (average cost per malaria, diarrhoea and suspected pneumonia cases appropriately diagnosed and treated according to protocol in a group of 100 eligible children, meaning that those that needed the treatment received it, and those that did not need the treatment did not receive it) and the ICERs. Due to the low numbers of children visiting a CBA in the Northern Region [19], the iCCM data of the Northern Region were excluded from this analysis.

**Table 6:** Cost-effectiveness for diagnosing and treating malaria per 100 eligible children from de societal perspective in 2014 (US$)

Variables	Volta Region		Northern Region	
	iCCM	CHPS	iCCM	CHPS
Number of eligible children for treatment	100	100		100
Number treated with ACT or quinine^a^	24	19		30
Number treated with prompt ACT or quinine	17	4		23
Number treated according to protocol (ACT or quinine)	28	30		35
Number treated according to protocol (prompt ACT or quinine)	21	15		27
Costs per 100 children (ACT or quinine)	US$119.04	US$182.30		US$244.57
Cost per child treated according to protocol (ACT or quinine)	US$4.25	US$6.12		US$7.09
Cost per child treated according to protocol (prompt ACT or quinine)	US$5.58	US$12.24		US$8.92
Incremental costs per 100 children (ACT or quinine)	US$63.26			
Incremental effect per 100 children (ACT or quinine)	2			
Incremental cost-effectiveness ratio (ACT or quinine)	iCCM is less costly and less effective			
Incremental costs per 100 children (prompt ACT or quinine)	US$63.26			
Incremental effect per 100 children (prompt ACT or quinine)	6			
Incremental cost-effectiveness ratio (prompt ACT or quinine)	iCCM is dominant			

^a^ *Source* Additional file 1

**Table 7:** Cost-effectiveness for diagnosing and treating diarrhoea per 100 eligible children from de societal perspective in 2014 (US$)

Variables	Volta Region^b^		Northern Region	
	iCCM	CHPS	iCCM	CHPS
Number of eligible children for treatment	100	100		100
Number treated with ORS (or referred)^a^	8	11		7
Number treated with ORS and zinc (or referred)	11	7		4
Number treated according to protocol (ORS or referred)	59	57		62
Number treated according to protocol (ORS and zinc)	54	43		59
Costs per 100 children (ORS)	US$6.84	US$83.20		US$38.88
Cost per 100 children (ORS and zinc)	US$9.78	US$47.54		US$19.44
Cost per child treated according to protocol (ORS or referred)	US$0.12	US$1.45		US$0.62
Cost per child treated according to protocol (ORS and zinc)	US$0.18	US$1.12		US$0.33
Incremental costs per 100 children (ORS or referred)	US$76.35			
Incremental effect per 100 children (ORS or referred)	2			
Incremental cost-effectiveness ratio (ORS or referred)	iCCM is dominant			
Incremental costs per 100 children (ORS or referred)	US$37.76			
Incremental effect per 100 children (ORS or zinc)	11			
Incremental cost-effectiveness ratio (ORS and zinc)	iCCM is dominant			

^a^ *Source* Additional file 2

^b^Appropriate treatment for diarrhoea under the iCCM strategy in the Volta Region includes those treated with ORS and zinc or referred for further management. The cost per treatment of those referred is included to allow comparison with CHPS

**Table 8:** Cost-effectiveness for diagnosing and treating suspected pneumonia per 100 eligible children from de societal perspective in 2014 (US$)

Variables	Volta Region^b^		Northern Region	
	iCCM	CHPS	iCCM	CHPS
Number of eligible children for treatment	100	100		100
Number treated with amoxicillin or cotrimoxazol^a^	15	16		23
Number treated according to protocol	72	72		73
Costs per 100 children	US$19.87	US$122.13		US$156.44
Cost per child treated according to protocol	US$0.274	US$1.693		US$2.13
Incremental costs per 100 children	US$102.25			
Incremental effect per 100 children	0			
Incremental cost-effectiveness ratio	iCCM is less costly and the same effective than CHPS			

^a^ *Source* Additional file 3

^b^Appropriate treatment for suspected pneumonia under the iCCM strategy in the Volta Region includes those treated with amoxicillin or referred for further management. The cost per treatment of those referred is included to allow comparison with CHPS

In the Volta Region, iCCM was more attractive than CHPS not only for the appropriate diagnosis and treatment of malaria, but also for the appropriate treatment of diarrhoea and suspected pneumonia. Note that even though the GFATM only provides ACT drugs for the iCCM strategy in the Volta Region, CBAs received training for the management of the three diseases [19]. If no drugs were available to treat a diarrhoea and pneumonia case (CBAs were provided with ORS, zinc and amoxicillin in 2013), CBAs were supposed to refer cases for further management, and this referral was also considered as appropriate treatment.

The average cost per malaria, diarrhoea and suspected pneumonia case appropriately diagnosed and treated was lower under the HBC than under the CHPS strategy. iCCM had lower costs than CHPS to diagnose and treat malaria, diarrhoea and suspected pneumonia cases while the effectiveness of both strategies was similar or slightly higher under the iCCM strategy (Tables 6, 7, 8).

The ICERs showed that HBC dominates CHPS for the prompt ACT or quinine treatment of malaria, for the treatment of diarrhoea with ORS or referred and for the treatment of diarrhoea with ORS and zinc (or referred). In the case of the management of suspected pneumonia, HBC was less costly and had the same effectiveness of CHPS. The management of malaria with ACT or quinine (including prompt and not prompt treatment) merits a further comment. Treating a malaria case under iCCM was less costly (US$119.04 versus US$182.30 were used to treat 100 children under the iCCM and CHPS strategy, respectively) but slightly less effective than under the CHPS strategy (28 and 30% of cases were treated according to protocol under iCCM and CHPS, respectively). Due to the small difference in effectiveness and the larger difference in cost, it can be concluded that iCCM was also cost-effective for the treatment of malaria with ACT or quinine when the promptness in treatment was not considered.

### Sensitivity analysis

The one-way sensitivity analysis explored how varying specific values influenced the average cost per case appropriately diagnosed and treated from the societal perspective in both strategies as well as the ICERs. For simplicity in presenting the data, only one ICER per disease was included, the one that better reflects the adherence to guidelines (prompt ACT for malaria, ORS and zinc for the management of diarrhoea and amoxicillin for the management of suspected pneumonia). iCCM in the Volta Region remained more cost-effective than CHPS for the diagnosis and treatment of malaria, diarrhoea and suspected pneumonia cases when using different facility costs, different discount rates and different iCCM and CHPS utilization. When iCCM effectiveness was reduced by 50%, iCCM remained cost-effective only for the treatment of diarrhoea (Table 9).

**Table 9:** Sensitivity to selected parameters of the cost-effectiveness ratio and the incremental cost-effectiveness ratio (ICER) for treating malaria, diarrhoea and suspected pneumonia in children under-five under iCCM and CHPS in the Volta Region

Indicators	iCCM	CHPS	iCCM	CHPS	iCCM	CHPS
	Average facility cost		More costly facility		Less costly facility	
Costs per 100 children (ACT or quinine)	119.0	182.3	141.1	234.0	97.0	130.6
Number treated according to protocol (prompt ACT or quinine)	21	15	21	15	21	15
Incremental cost-effectiveness ratio (prompt ACT or quinine)	iCCM dominates		iCCM dominates		iCCM dominates	
Cost per 100 children (ORS and zinc)	9.8	47.5	11.2	64.4	8.3	30.7
Number treated according to protocol (ORS and zinc)	54	43	54	43	54	43
Incremental cost-effectiveness ratio (ORS and zinc)	ICCM dominates		iCCM dominates		iCCM dominates	
Costs per 100 children (amoxicillin)	19.9	122.1	20.5	168.9	19.1	75.6
Number treated according to protocol (amoxicillin)	72	72	72	72	72	72
Incremental cost-effectiveness ratio (amoxicillin)	iCCM less costly/same effectiveness		iCCM less costly/same effectiveness		iCCM less costly/same effectiveness	

	3% discount rate		5% discount rate		7% discount rate	
Costs per 100 children (ACT or quinine)	119.04	182.30	121.20	187.66	127.68	202.60
Number treated according to protocol (prompt ACT or quinine)	21	15	21	15	21	15
Incremental cost-effectiveness ratio (prompt ACT or quinine)	iCCM dominates		iCCM dominates		iCCM dominates	
Cost per 100 children (ORS and zinc)	9.78	47.54	9.78	48.46	9.89	49.84
Number treated according to protocol (ORS and zinc)	54	43	54	43	54	43
Incremental cost-effectiveness ratio (ORS and zinc)	iCCM dominates		iCCM dominates		iCCM dominates	
Costs per 100 children (amoxicillin)	19.87	122.13	19.87	124.59	19.87	127.87
Number treated according to protocol (amoxicillin)	72	72	72	72	72	72
Incremental cost-effectiveness ratio (amoxicillin)	iCCM less costly/same effectiveness		iCCM less costly/same effectiveness		iCCM less costly/same effectiveness	

	Average utilization		Higher utilization		Lower utilization	
Costs per 100 children (ACT or quinine)	119.04	182.30	110.40	166.21	144.00	226.91
Number treated according to protocol (prompt ACT or quinine)	21	15	21	15	21	15
Incremental cost-effectiveness ratio (prompt ACT or quinine)	iCCM dominates		iCCM dominates		iCCM dominates	
Cost per 100 children (ORS and zinc)	9.78	47.54	8.67	43.48	12.78	50.23
Number treated according to protocol (ORS and zinc)	54	43	54	43	54	43
Incremental cost-effectiveness ratio (ORS and zinc)	iCCM dominates		iCCM dominates		iCCM dominates	
Costs per 100 children (amoxicillin)	19.87	122.13	14.64	112.13	36.46	128.85
Number treated according to protocol (amoxicillin)	72	72	72	72	72	72
Incremental cost-effectiveness ratio (amoxicillin)	iCCM less costly/same effectiveness		iCCM less costly/same effectiveness		iCCM less costly/same effectiveness	

	Average effectiveness		Higher effectiveness		Lower effectiveness	
Costs per 100 children (ACT or quinine)	119.04	182.30	111.86	178.28	88.40	186.13
Number treated according to protocol (prompt ACT or quinine)	21	15	31	15	11	15
Incremental cost-effectiveness ratio (prompt ACT or quinine)	iCCM dominates		iCCM dominates		iCCM is less costly and less effective	
Cost per 100 children (ORS and zinc)	9.78	47.54	6.82	47.15	14.40	47.87
Number treated according to protocol (ORS and zinc)	54	43	59	43	49	43
Incremental cost-effectiveness ratio (ORS or zinc)	iCCM dominates		iCCM dominates		iCCM dominates	
Costs per 100 children (amoxicillin)	19.87	122.13	19.55	121.97	19.97	122.30
Number treated according to protocol (amoxicillin)	72	72	80	72	65	72
Incremental cost-effectiveness ratio (amoxicillin)	iCCM dominates		iCCM dominates		iCCM is less costly and less effective	

## Discussion

Before the adoption of the iCCM and CHPS strategies in the Ghana Health System, RCT were conducted to assess their efficacy in Ghana [13, 16]. In addition, another study was conducted to assess the cost-effectiveness of two strategies of HBC [38], although without presenting the cost per fever treated under the standard care. The current evaluation used data from an observational study to analyse the cost-effectiveness of iCCM and CHPS strategies after 2 and 8 years of iCCM implementation in the Volta and the Northern Regions respectively for the treatment of malaria, diarrhoea and suspected pneumonia in children under-five. Results from this evaluation may be used to improve iCCM and CHPS implementation and to guide policy makers in the discussion about long term financing of iCCM.

This study brings more light to the scarce evidence on iCCM cost-effectiveness. Three comparable studies were found on the cost-effectiveness of malaria HBC versus standard care conducted in Ghana [38], Uganda [39] and Zambia [40]. No studies were found on the cost-effectiveness of treatment of diarrhoea and/or pneumonia delivered through iCCM. A systematic review that looked at the effect of community-case management of pneumonia in Africa concluded that there is a lack of evidence on its efficacy [41].

Results from the current study showed that iCCM in the Volta Region was more cost effective than CHPS for the management of malaria, diarrhoea and suspected pneumonia in children under-five, even after the sensitivity analysis modifying health facility costs, discount rates and service utilization. However, if the iCCM effectiveness was reduced by 50%, HBC remained less costly but less effective than CHPS and therefore, less cost-effective. These results are coherent with the three studies mentioned above, which concluded that iCCM was more cost-effective than the standard care (defined as the care received from a health facility). The RCT conducted in the south of Ghana [38] and the Markov modelling using data from Uganda [42] looked at the cost per DALY averted. The study conducted in Zambia [40] used case appropriately diagnosed and treated as the effectiveness measure, concluding that malaria iCCM was more cost-effective than the standard care. Some differences however can be seen between the Zambia study and the current study: the Zambia analysis was done from the provider perspective, used registries instead of survey data to assess effectiveness, and RDTs were used within the iCCM strategy. Excluding household costs could bias results in favour of CHPS (the current study showed that household costs were higher when visiting a CHPS than a CBA). Using registries instead of surveys to collect effectiveness data could also introduce differences: registers might suffer from incomplete reporting and survey data might suffer from recall bias and misinterpretation of symptoms. Using an RDT for malaria diagnosis in iCCM could increase iCCM cost (due to staff time and laboratory costs) but drug costs might be reduced (if CHW adhere to RDT results).

The cost-effectiveness analysis was not conducted in the Northern Region due to the low numbers of carers visiting a CBA. However, the cost analysis showed that iCCM programme costs in the Northern Region were higher than those in the Volta Region and higher than CHPS costin the Volta and in Northern Region, particularly due to the higher number of CBAs registered doing few preventive and curative activities.

Long term financing is a cornerstone of iCCM, often financed by health partners without a clear sustainability strategy. However, if iCCM is an effective and cost-effective intervention, other financial plans must be considered. In Ghana, the NHIS pays health providers for services included in the insurance benefit package. A condition to include a service into the NHIS benefit package is its cost-effectiveness. The current study showed that the curative component of the iCCM strategy in the Volta Region was more cost-effective than CHPS. In addition, iCCM contributes to health equity as it was able to reach the poorest in the Volta Region [19], and it was associated with disease knowledge and healthy behaviours in the Northern Region.

All these points should make the iCCM strategy “attractive” to be included in the NHIS benefit package. However, the NHIS must have the money to finance this strategy without increasing the already high burden of the insurance expenditure. In the period 2009–2013 the NHIS expenditures exceeded revenues [43–45]. The 2014 and 2015 reports were not available. Revising the NHIS benefit package as planned in 2014 [46] and considering iCCM strategy among the possible benefits based on the results of this study could be a positive way forward. From the point of view of implementation, it is important to reflect on how the NHIS could reimburse iCCM activities. CHPS are facilities registered under the NHIS. CBAs curative activities reported to CHPS could be reimbursed by the NHIS as activities related to CHPS, which is informally happening already in some districts. If the preventive component of iCCM is also considered (which is likely to be cost-effective when compared to CHPS-because of its lower costs and higher effect on disease knowledge and health behaviour, although not assessed here), some IEC activities, particularly those done in partnership with CHPS under Community, IMCI could also be reimbursed by the NHIS. This could help the sustainability of iCCM, retain CBAs and improve the quality of the intervention. However, control measures must be put in place to avoid reporting false activity. Another issue to be considered is the acceptability, particularly of the CHPS and the NHIS. An acceptability study should be conducted to assess the perceptions of different stakeholder.

### Limitations of the study

The effectiveness measure in this study was obtained from a cross sectional study. Effectiveness data in economic evaluations are often recommended to be from RCT although there are limitations such as the comprehensiveness (only one source of data) and the short time horizons. To overcome these limitations, there is now a tendency to use data from systematic reviews or even better, from decision analytic modelling [33]. This is true if one wants to prove the efficacy and efficiency of a new intervention. But if one wants to evaluate the effectiveness and efficiency of a proven intervention in a real life routine setting, meaning evaluating its costs and effects during actual implementation as in an ex-post HTA, then data from observational studies are more relevant [3, 4, 33] and have been used in several studies in the UK, Asia and Africa [40, 47–51].

The effectiveness measure used was ‘case appropriately diagnosed and treated’ as a proxy indicator of child mortality. Even though intermediate outputs are admissible in cost-effectiveness analysis (although it is better to use a final health outcome) [33], this measure might bring difficulties when summarizing results from each of the three diseases evaluated. However, the fact that results were similar among the three diseases evaluated (iCCM was more cost-effective for the treatment of malaria, diarrhoea and suspected pneumonia) made the interpretation easier. In addition, the effectiveness measure used does not have a threshold to define whether an intervention is cost-effective or not. Ideally, the government should be questioned about their willingness to pay per extra case appropriately treated. However, as the iCCM strategy was less costly than CHPS, this limitation was solved. Case appropriately diagnosed and treated measures the quality of care given as it refers to adherence to guidelines. However, it does not consider the outcome of death or disability (although the cost for visiting a second provider was considered in case the child did not recover). Even it would not be expected that not considering death and disability might influence the results—no deaths or disability were reported—using DALYs or QALYs might have solved the issue of the different end points of treatment. In addition, the use of DALYs/QALYs might have allowed a comparison of the study results with other diseases and studies.

Case appropriately diagnosed in this study refers to adherence to guidelines and not to adherence to microscopy results which is the gold standard method for diagnosing malaria (which cannot be performed at this community level). This means that the appropriate diagnosis of malaria under the iCCM strategy refers to a child with fever, while in the CHPS refers to a child with a positive RDT or a child with fever if not RDT was performed. Although some fever cases might be misclassified as malaria cases, this is something to consider when revising the iCCM guidelines but not in this current study (which objective is to assess the iCCM and CHPS implementation and the adherence to guidelines).

Data from the cross-sectional study brought relevance to the evaluation but also brought limitations. Carers of sick children chose their providers based on different criteria (such as proximity, trust, availability or perceived severity of disease), reflecting the actual utilization of the services. This self-selection also affected the cost-effectiveness analysis: few carers of sick children chose to visit a CBA in the Northern Region. Due to these low numbers of carers visiting a CBA in the Northern Region it was not possible to conduct the cost-effectiveness analysis. Self-selection is a factor to be considered when addressing cost- effectiveness. Children would have been taken to a CHPS if their disease was perceived to be more severe by their carer, reducing the cost-effectiveness of CHPS compared to iCCM. However, this study aimed to assess the cost-effectiveness of iCCM in the routine system, and therefore, this self-selection should be considered as part of the system and not as a source of bias.

Sources of facility costs came from data of two CHPS in the Volta Region and two in the Northern Region. Although the analysis took into consideration average costs, and higher and lower facility cost for the sensitivity analysis, it would have been better to have at least one more CHPS in each region to have a better representation of CHPS costs. Unfortunately, it was not possible to add another CHPS to the cost analysis.

Finally, different epidemiological burden would not be expected to influence results, as the target population was children with symptoms. However, finding these children when doing the data collection was easier in the Northern Region as the prevalence of the three diseases was higher.

## Conclusions

This study added to the scarce evidence on the iCCM cost-effectiveness. iCCM for treating malaria, diarrhoea and suspected pneumonia in children under-5 years of age was more cost-effective than treatment through CHPS in the Volta Region. High programme costs in addition to low curative and preventive activities made the HBC strategy in the Northern Region more costly than in the Volta Region. A revision of the iCCM strategy in the Northern Region is needed to improve its cost-effectiveness. This study supports the need for a long-term financing strategy, such as inclusion of iCCM in the NHIS benefit package. However, a revision of the NHIS benefit package and an acceptability study must be conducted before making such a decision. Then, the appropriateness of a pilot study including iCCM services in the NHIS could be considered.

## Additional files

**Additional file 1.** Effect and cost for malaria diagnosis and treatment under HBC and CHPS strategy in the Volta and the Northern Regions.

**Additional file 2.** Effect and cost for diarrhoea diagnosis and treatment under HBC and CHPS strategy in the Volta and the Northern Regions.

**Additional file 3.** Effect and cost for suspected pneumonia diagnosis and treatment under HBC and CHPS strategy in the Volta and the Northern Regions.

## Abbreviations

ARI: acute respiratory infection
CBAs: community-based agents
CHPS: community-based health planning services
CHW: community health workers
DALYs: disability adjusted life years
GHS: Ghana health service
HTA: health technology assessment
HBC: home-based care
iCCM: integrated community case management
ICER: incremental cost-effectiveness ratio
IMCI: integrated management of childhood illness
NMCP: National malaria control programme
QALY: quality adjusted life years
RCT: randomized controlled trial
