# Supplementary material for: Cost-effectiveness analysis of the national implementation of integrated community case management and community-based health planning and services in Ghana for the treatment of malaria, diarrhoea and pneumonia
Source: Malar J. 2017 Jul 5;16:277. doi: 10.1186/s12936-017-1906-9 (PMC5498878; doi:10.1186/s12936-017-1906-9)
Supplement: Supplementary file 1 — Additional file 1. Effect and cost for malaria diagnosis and treatment under HBC and CHPS strategy in the Volta and the Northern Regions. [file 12936_2017_1906_MOESM1_ESM.docx]

| **Additional file 1. Effect and cost for malaria diagnosis and treatment under HBC and CHPS strategy in the Volta and the Northern Regions** | | | | |
| --- | --- | --- | --- | --- |
| **MALARIA** | | | | |
|  | **Volta Region** | | **Northern Region** | |
| **Variables** | **iCCM** | **CHPS** | **iCCM** | **CHPS** |
| Number of eligible children for treatment | **75** | **47** | **6** | **197** |
| Number of non-complicated malaria cases | 70 | 40 | 5 | 183 |
| Number of non-complicated malaria cases treated with ACT | 17 | 7 | 1 | 26 |
| Number of non-complicated malaria cases treated with ACT or quinine | 17 | 8 | 1 | 57 |
| Number of non-complicated malaria cases treated with prompt ACT or quinine | 12 | 1 | 1 | 43 |
| Number of no malaria cases treated with antimalarial | 1 | 1 | 0 | 3 |
| Number of no malaria not treated with antimalarial | 4 | 6 | 1 | 11 |
| **Number of cases treated according to protocol (ACT or quinine)** | **21** | **14** | **2** | **68** |
| **Number of cases treated according to protocol (prompt ACT or quinine)** | **16** | **7** | **2** | **54** |
| % of cases treated according to protocol (ACT or quinine) | 0.28 | 0.30 | 33.33 | 34.52 |
| % of cases treated according to protocol (prompt ACT or quinine) | 0.21 | 0.15 | 33.33 | 27.41 |
| Cost per malaria treatment* | 4.96 | 9.52 | 9.37 | 8.03 |

* Source: Table 5
