# Supplementary material for: Cost-effectiveness analysis of the national implementation of integrated community case management and community-based health planning and services in Ghana for the treatment of malaria, diarrhoea and pneumonia
Source: Malar J. 2017 Jul 5;16:277. doi: 10.1186/s12936-017-1906-9 (PMC5498878; doi:10.1186/s12936-017-1906-9)
Supplement: Supplementary file 2 — Additional file 2. Effect and cost for diarrhoea diagnosis and treatment under HBC and CHPS strategy in the Volta and the Northern Regions. [file 12936_2017_1906_MOESM2_ESM.docx]

| **Additional file 2. Effect and cost for diarrhoea diagnosis and treatment under HBC and CHPS strategy in the Volta and the Northern Regions** | | | | |
| --- | --- | --- | --- | --- |
| **DIARRHOEA** | | | | |
|  | **Volta Region** | | **Northern Region** | |
| **Variables** | **iCCM** | **CHPS** | **iCCM** | **CHPS** |
| **Number of eligible children for treatment** | **90** | **61** | **8** | **228** |
| Number of diarrhoea cases | 38 | 31 | 4 | 86 |
| Number of diarrhoea cases treated with ORS (or referred) | 4 | 6 | 1 | 8 |
| Number of diarrhoea cases treated with zinc (or referred) | 6 | 6 | 1 | 3 |
| Number of diarrhoea cases treated with ORS and zinc | 3 | 0 | 0 | 0 |
| Number of no diarrhoea cases treated with ORS | 3 | 1 | 0 | 8 |
| Number of no diarrhoea cases treated with zinc | 4 | 3 | 0 | 0 |
| Number of no diarrhoea cases treated with ORS or zinc | 7 | 4 | 0 | 8 |
| Number of no diarrhoea cases not treated with ORS | 49 | 29 | 4 | 134 |
| Number of no diarrhoea cases not treated with zinc | 48 | 27 | 4 | 142 |
| Number of no diarrhoea cases not treated with ORS or zinc | 46 | 26 | 4 | 134 |
| **Number of cases treated according to protocol (ORS and zinc)** | 49 | 26 | 4 | 134 |
| **Number of cases treated according to protocol (ORS)** | 53 | 35 | 5 | 142 |
| **Number of cases treated according to protocol (zinc)** | 54 | 33 | 5 | 145 |
| % of cases treated according to protocol (ORS and zinc) | 0.54 | 0.43 | 0.50 | 0.59 |
| % of cases treated with ORS | 0.59 | 0.57 | 0.63 | 0.62 |
| % of cases treated with zinc | 0.60 | 0.54 | 0.63 | 0.64 |
| Cost per diarrhoea treatment (giving zinc or ORS)* | 0.88 | 7.25 | 8.36 | 5.54 |

* Source: Table 5
