# Supplementary material for: Cost-effectiveness analysis of the national implementation of integrated community case management and community-based health planning and services in Ghana for the treatment of malaria, diarrhoea and pneumonia
Source: Malar J. 2017 Jul 5;16:277. doi: 10.1186/s12936-017-1906-9 (PMC5498878; doi:10.1186/s12936-017-1906-9)
Supplement: Supplementary file 3 — Additional file 3. Effect and cost for suspected pneumonia diagnosis and treatment under HBC and CHPS strategy in the Volta and the Northern Regions. [file 12936_2017_1906_MOESM3_ESM.docx]

| **Additional file 3. Effect and cost for suspected pneumonia diagnosis and treatment under HBC and CHPS strategy in the Volta and the Northern Regions** | | | | | | |
| --- | --- | --- | --- | --- | --- | --- |
| **SUSPECTED PNEUMONIA** |  | | | | |  |
|  | **Volta Region** | | | **Northern Region** | |  |
| **Variables** | **iCCM** | **CHPS** | **iCCM** | | **CHPS** | |
| **Number of eligible children for treatment** | **87** | **61** | **7** | | **228** | |
| Number of suspected pneumonia cases | 25 | 9 | 1 | | 15 | |
| Number of suspected pneumonia cases that received amoxicillin (or referred) | 7 | 1 | 0 | | 3 | |
| Number of suspected pneumonia cases that received amoxicillin or cotrimoxazol (or referred) | 7 | 1 | 0 | | 4 | |
| Number of no suspected pneumonia treated with amoxicillin or cotrimoxazol | 6 | 9 | 0 | | 50 | |
| Number of no suspected pneumonia not treated with amoxicillin or cotrimoxazol | 56 | 43 | 6 | | 163 | |
| **Number of cases treated according to protocol** | **63** | **44** | **6** | | **167** | |
| % of cases treated according to protocol | 0.72 | 0.72 | 0.86 | | 0.73 | |
| Cost per suspected pneumonia treatment* | 1.33 | 7.45 | 8.50 | | 6.73 | |

* Source: Table 5
